# Deformable devices with integrated functional nanomaterials for wearable electronics

**DOI:** 10.1186/s40580-016-0062-1

**Published:** 2016-03-15

**Authors:** Jaemin Kim, Jongsu Lee, Donghee Son, Moon Kee Choi, Dae-Hyeong Kim

**Affiliations:** 1 Center for Nanoparticle Research, Institute for Basic Science (IBS), Seoul, 151-742 Republic of Korea; 2 School of Chemical and Biological Engineering, Institute of Chemical Processes, Seoul National University, Seoul, 151-742 Republic of Korea

**Keywords:** Silicon nanomembrane, Functional nanomaterials, Flexible electronics, Stretchable electronics, Wearable electronics

## Abstract

As the market and related industry for wearable electronics dramatically expands, there are continuous and strong demands for flexible and stretchable devices to be seamlessly integrated with soft and curvilinear human skin or clothes. However, the mechanical mismatch between the rigid conventional electronics and the soft human body causes many problems. Therefore, various prospective nanomaterials that possess a much lower flexural rigidity than their bulk counterparts have rapidly established themselves as promising electronic materials replacing rigid silicon and/or compound semiconductors in next-generation wearable devices. Many hybrid structures of multiple nanomaterials have been also developed to pursue both high performance and multifunctionality. Here, we provide an overview of state-of-the-art wearable devices based on one- or two-dimensional nanomaterials (e.g., carbon nanotubes, graphene, single-crystal silicon and oxide nanomembranes, organic nanomaterials and their hybrids) in combination with zero-dimensional functional nanomaterials (e.g., metal/oxide nanoparticles and quantum dots). Starting from an introduction of materials strategies, we describe device designs and the roles of individual ones in integrated systems. Detailed application examples of wearable sensors/actuators, memories, energy devices, and displays are also presented.

## Introduction

In the rapid technology development of low-power silicon electronics, light-emitting diodes (LEDs) fabricated on unconventionally shaped substrates, high-capacity lithium-ion batteries, and various wearable electronic devices such as smart glasses, watches, and lenses have been unveiled both in academic journals and on the market. In spite of their superb performance, wearable form factors, and compact sizes, challenges remain mainly owing to their large thickness and mechanical rigidity, which result in a mechanical mismatch between the device and the skin, and thereby discomfort, a low signal-to-noise ratio, and measurement errors [1]. In this regard, achieving mechanical deformability of the wearable electronic/optoelectronic devices while maintaining high performances has been a major research goal [2–6].

One promising strategy is to replace the rigid electronic materials (e.g., silicon wafer) with flexible nanomaterials (e.g., silicon nanomembrane (SiNM) [7–11], carbon nanotubes (CNTs) [12–14], graphene (GP) [1, 15, 16], and organic nanomaterials [17, 18]). The electronic properties of the SiNM (down to tens of nanometers) remain the same as the bulk silicon wafer [19], but its bendability dramatically increases owing to the reduced thickness [5]. SiNM-based devices outperform their competitors including low-temperature polycrystalline silicon (LTPS) and organic devices by virtue of their high electron mobility [20]. However, SiNM based device might have issues in the high cost and complicated fabrication processes. Meanwhile, carbon nanomaterials (e.g., CNTs and GP) [21, 22] have been getting attentions as next-generation semiconducting nanomaterials. The mobility of single-walled CNTs (SWNTs) and exfoliated GP have been reported up to 100,000 [21] and 230,000 cm^2^ V^−1^ s^−1^ [22], respectively, which are higher than that of single-crystal silicon. Their ultrathin thickness enables them to be seamlessly integrated in wearable devices while achieving the transparency [23–25]. The mass production, device performance, and fabrication processes of these carbon nanomaterials, however, have many remaining challenges for commercial device applications [26]. Organic nanomaterials such as organic nanowires/nanofibers also have recently utilized as electric materials for fabricating complementary metal–oxide–semiconductor (CMOS) circuits [27] and wearable power generators [28, 29]. Intrinsic deformability of organic nanomaterials, solution processability, and low cost make them promising for wearable devices [27]. However, their low electrical performances should be resolved for its widespread applications [17].

**Fig. 1:**
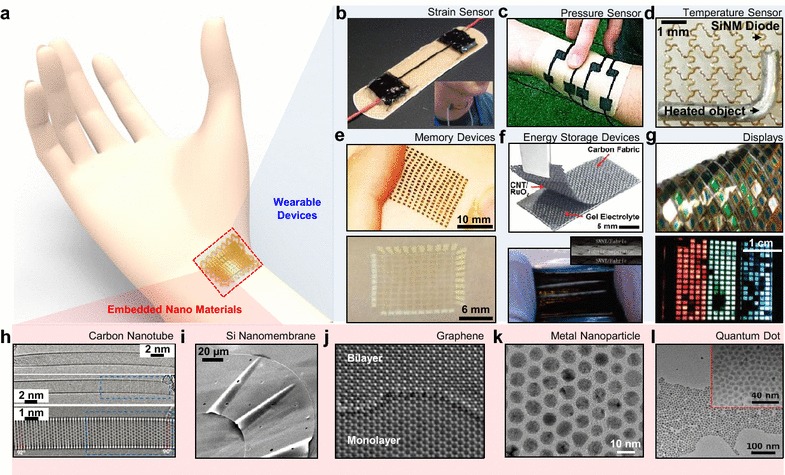
Overview of wearable devices with integrated nanomaterials. **a** Schematic of a wearable device mounted on human skin. **b**–**g** Optical images of representative wearable devices consisting of functional nanomaterials: **b** strain sensor, **c** pressure sensor, **d** temperature sensor, **e** memory arrays, **f** energy storage devices; and **g** displays. (**b**–**g** Reproduced with permission from **b** Ref. [13], © 2011, Nature Publishing Group; **c** Ref. [47], © 2014, WILEY–VCH Verlag GmbH & Co. KGaA, Weinheim; **d** Ref. [7], © 2014, Nature Publishing Group; **e** Refs. [48] and [41], © 2015, American Chemical Society and © 2014, Nature Publishing Group; **f** Ref. [45] and [49], © 2014, WILEY–VCH Verlag GmbH & Co. KGaA, Weinheim and © 2010, American Chemical Society; **g** Refs. [51] and [50], © 2009, Nature Publishing Group and © 2013, Nature Publishing Group). **h**–**l** Transmission/scanning electron microscopy (TEM/SEM) images of representative functional nanomaterials integrated into wearable devices: **h** CNTs, **i** an SiNM, **j** GP, **k** metal nanoparticles, and **l** quantum dots. (**h**–**l** Reproduced with permission from **h** Ref. [52], © 2011, Nature Publishing Group; **i** Ref. [7], © 2014, Nature Publishing Group; **j** Ref. [53], © 2011, Nature Publishing Group; **k** Ref. [46], © 2015, WILEY–VCH Verlag GmbH & Co. KGaA, Weinheim; **l** Ref. [44], © 2015, Nature Publishing Group)

Another approach to achieve both high performance and multifunctionality is to utilize hybrids of nanomaterials [30–36]. Functional hybrid nanomaterials often exhibit substantially different physical, mechanical, magnetic, chemical, and optical properties compared to their individual and/or bulk counterparts [37–40]. By integrating different functional nanomaterials, the performance of wearable devices can be dramatically improved and/or diversified [1, 7, 41–46]. For the realization of this goal, the type, size, thickness, and concentration of the nanomaterials should be carefully designed [46]. In the following, we summarize recently reported wearable sensors/actuators [7, 13, 47], memories [41, 48], energy storage devices [49], and displays [50, 51] that exploit various nanomaterials [7, 44, 46, 52, 53] and their hybrids (Fig. 1). We also describe the roles of each nanomaterial in specific devices, improved device functions, their system integrations, and provide brief perspectives on future research directions.

## Review

### Wearable sensors/actuators

Wearable sensors/actuators have recently attracted considerable interest because of their mobile healthcare [54] and virtual reality applications [55]. Sensors/actuators worn on the body, in particular, have drawn attention for the continuous and accurate monitoring of physiological (e.g., motion [1, 47] and temperature [56, 57]) and electrophysiological (e.g., electrocardiograms [58, 59] and electromyograms [60, 61]) signals and appropriate instant feedback on them [1], which are important for point-of-care medical diagnostics and therapy. This section describes representative wearable sensors/actuators based on functional nanomaterials and their application examples in healthcare and human–machine interfaces.

#### SiNM-based sensors

Deformability, which is one of the key characteristics of wearable electronics, can be achieved by making inorganic materials (i.e., silicon) as thin as possible, down to the nanometer scale (i.e., nanomembrane) [5]. SiNM can be fabricated through several processes. One easy fabrication method is to remove the buried oxide of a silicon-on-insulator (SOI) wafer and pick the top part up or to etch the bottom silicon of the SOI wafer and use the remaining top part [7]. The obtained SiNM can be located in the desired position of the designed layout by using previously reported transfer printing techniques. SiNM maintains the high carrier mobility [20] and intrinsic piezoresistivity [7] of the bulk monocrystalline silicon, while having a high flexibility, which enables diverse wearable electronics applications.

**Fig. 2:**
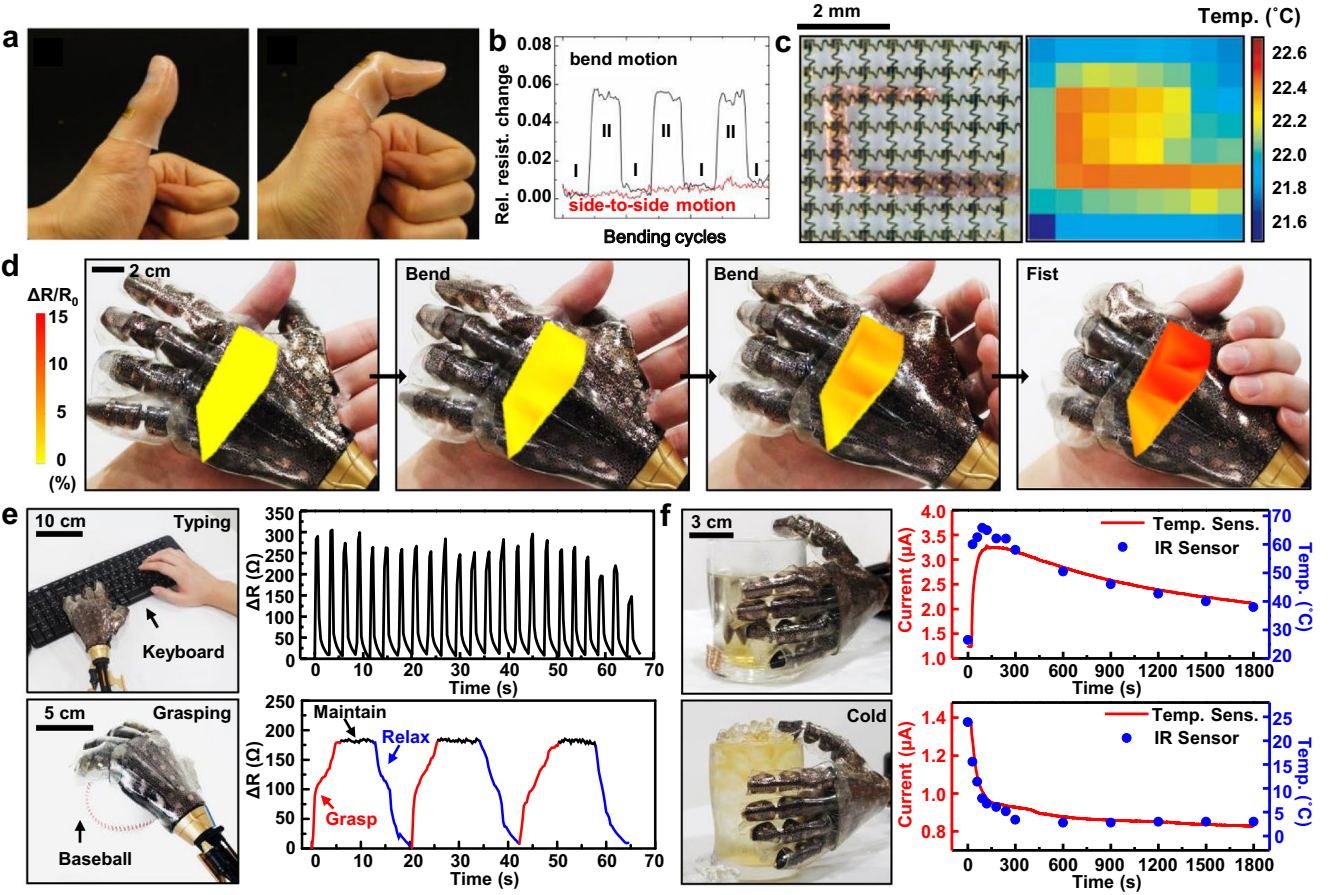
SiNM-based wearable sensors. **a** SiNM-based strain gauge array on a polymer-based finger tube mounted on a relaxed (*left*) and bent thumb (*right*). **b** Relative change in the resistance of the SiNM strain gauge according to the bending (*black*) and side-to-side (*red*) motion of the thumb. (**a**, **b** Reproduced with permission from Ref. [8], © 2012, IOP Science). **c** SiNM-diode-based wearable temperature sensor located on a heated metal element (*left*) and the measured temperature distribution (*right*). **d** SiNM strain gauge array integrated onto prosthetic skin, which measures the strain distribution when during handshaking. (**c**, **d** Reproduced with permission from Ref. [57], © 2013, Nature Publishing Group). **e** SiNM pressure sensor integrated onto prosthetic skin, which measures the applied pressure when the wearer is typing with a keyboard (*top right*) and grasping a ball (*bottom left*), and the corresponding measured change in the resistance of the SiNM pressure sensor (*right*). **f** SiNM temperature sensor integrated onto prosthetic skin, which measures the changes in the temperature of hot (*top*) and cold (*bottom*) glass cups. (**d**–**f** Reproduced with permission from Ref. [7], © 2014, Nature Publishing Group)

For instance, multiplexing through SiNM transistors integrated into the flexible high-density electrode array achieves the real-time analysis of electrophysiological signals over a large area of the brain [10] and heart [62] surface. SiNM strain gauges integrated onto polymeric substrates are applied as wearable motion sensors thanks to their high piezoresistive sensitivity [7, 8]. Figure 2a shows images of a SiNM strain gauge array integrated with a finger tube that conforms to the thumb. The bending motion of the thumb applies a tensile stress to the SiNM strain gauges, and their resistance increases accordingly without any hysteresis (Fig. 2b). Multiplexing by SiNM p–i–n junction diodes is also advantageous for constructing a wearable high-spatial-resolution temperature sensor array. Figure 2c depicts an 8 × 8 p–i–n junction diode array located on a heated Cu element (left) and its measured temperature distribution (right). The rectifying characteristics of silicon diodes enable each cell to be individually addressable with the minimum number of wires and crosstalk, achieving a high spatial resolution. The ultrathin dimensions of the sensor array facilitate not only conformal contacts with the target surface but also a fast response time by virtue of its extremely low thermal mass.

By combining the SiNM strain gauge, pressure sensor, and temperature sensor array in a single platform, a skin-like device conformally mounted onto a prosthetic arm is demonstrated. The SiNM strain gauge array monitors the change in the strain distribution according to the clenching motion of the prosthetic hand (Fig. 2d). Similarly, the SiNM pressure sensor measures the applied pressure when typing with a keyboard (Fig. 2e, top) and grasping a baseball (Fig. 2e, bottom). The SiNM temperature sensor mounted on the prosthetic skin distinguishes different surface temperatures (Fig. 2f). Although these SiNM sensors exhibit a high potential for various wearable sensing applications, there are cost issues to be addressed for the development of commercial products.

#### CNT-based wearable sensors

**Fig. 3:**
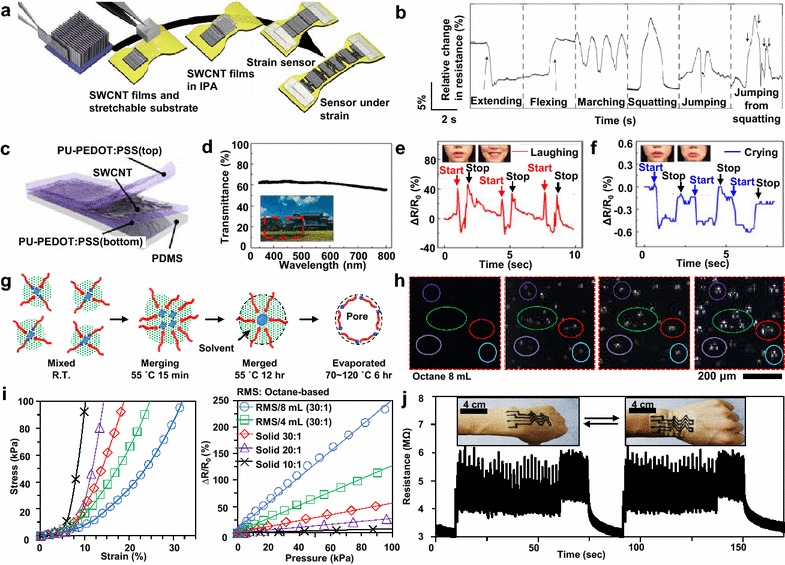
CNT-based wearable sensors. **a** Schematic of the fabrication process of the aligned CNT strain sensor. **b** Relative change in the resistance of the strain sensor patched onto the knee depending on his/her specific movements. (**a**–**b** Reproduced with permission from Ref. [13], © 2011, Nature Publishing Group). **c** Schematic exploded view of the transparent patchable strain gauge. **d** Transmittance of the strain gauge depending on the wavelength and a photograph showing the device placed on a picture (*inset*). **e**, **f** Relative change in the resistance of the strain sensor patched onto the face while **e** he/she is laughing and **f** crying. (**c–f** Reproduced with permission from Ref. [14], © 2015, American Chemical Society). **g** Schematics of the pore generation process according to the heat treatment conditions. **h** Successive images of the ECR under heat treatment, showing the gradually forming pores inside. **i** Stress–strain curves of various types of ECRs, obtained by deforming them and measuring the strain and applied stress (*left*), and the relative change in the resistance of the ECRs with respect to the applied pressure (*right*). **j** Change in the resistance of the wearable porous ECRs according to the repetitive bending and relaxing motions of the wrist. (**g–j** Reproduced with permission from Ref. [47], © 2014, WILEY–VCH Verlag GmbH & Co. KGaA, Weinheim)

The macroscopic form of CNTs in most devices is either their aligned arrays or random networks. Hata et al. [12] developed a synthesis method for ultra-long vertically aligned CNTs using the water-assisted chemical vapor deposition (CVD) process. The vertically aligned CNTs could be selectively grown on a patterned catalyst layer and transferred onto a stretchable substrate for device applications such as a strain sensor (Fig. 3a) [13]. In this strain measurement, the CNT film deforms as the substrate is stretched and its resistance increases. This relative change in the resistance according to the applied strain can be used for human-motion detection. When the sensor is attached to a human knee, the change in the resistance exhibits variations corresponding to the wearer’s motion (Fig. 3b). Although vertical CNTs are densely aligned similar to a forest and therefore have a dark color, randomly oriented CNT networks are relatively transparent, particularly at reduced CNT concentrations [14]. Figure 3c shows a schematic of a transparent patch-type strain sensor using random-network CNTs integrated with a conducting polymer. By virtue of its optical transparency (62 %), it was inconspicuously patched onto a human face and successfully distinguished facial motions (Fig. 3d–f).

CNTs are also excellent nanoscale filler materials owing to their small size with good dispersion and exceptional electrical and physical properties [63, 64]. In this regard, electrically conductive rubber (ECR), which is a composite of CNTs and elastomeric polymers, is prepared and used for a wearable mechanical sensor [47]. To enhance the sensitivity, nanopores and micropores are introduced into the ECR, thereby increasing its piezoresistivity and maximizing the locally induced strain when deformed [47]. Figure 3g shows a representative method for introducing pores with a uniform size and distribution in the ECR. The key idea of this method is to use a reverse micelle solution (RMS) comprising an emulsifier, deionized (DI) water, and an organic solvent. In accordance with careful sequential heat treatments, the migration and merging of the reverse micelles and subsequent pore generations occur (Fig. 3h). A larger porosity and lower elastic modulus are achieved if a larger amount of solvent is included in the RMS, thereby resulting in a higher pressure sensitivity (Fig. 3i). An ECR-based strain gauge fabricated on a medical bandage by using ink-jet printing forms a conformal contact with the human wrist and successfully monitors wrist motions. Although sensors based on CNT networks/composites are relatively cheap, especially those that are solution-processed, and mechanically compatible when worn on the human body, several issues such as a slow response time, a large area uniformity, and the hysteresis and drift of signals still need to be solved.

#### Wearable sensors/actuators based on nanomaterial hybrids

**Fig. 4:**
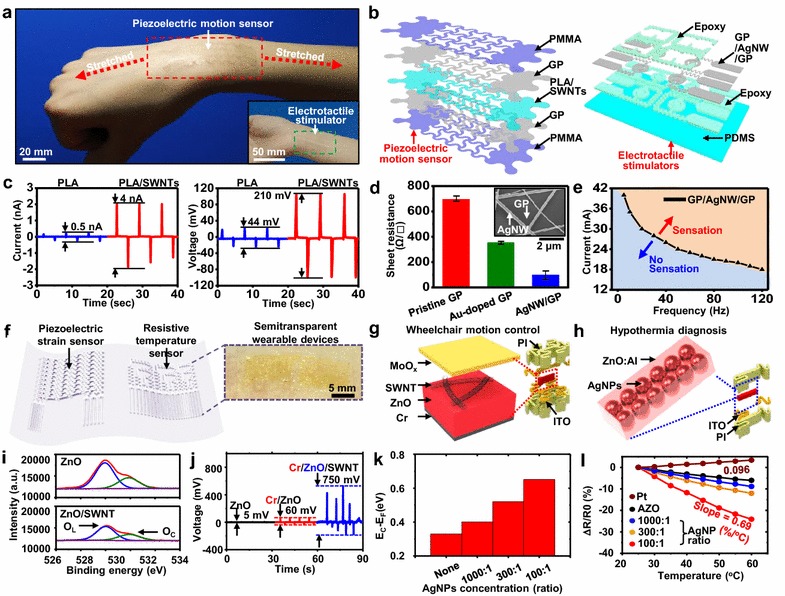
Wearable devices with performance-enhancing nanomaterials. **a** Photographs showing a transparent wearable motion sensor based on a GP heterostructure and the wearable electrotactile stimulator mounted onto human skin. **b** Schematic exploded structure of the transparent wearable motion sensor (*left*) and electrotactile stimulator (*right*). **c** Output current (*left*) and voltage (*right*) of the wearable motion sensor consisting of PLA (*blue*) and PLA/SWNTs (*red*) according to the bending and relaxing motions of the device. **d** Sheet resistance of pristine GP (*red*), GP doped with an AuCl_3_ solution (*green*), an AgNW/GP composite (*blue*), and its SEM image (*inset*). **e** Minimum required current for the wearer to sense stimulation with respect to the stimulation frequency. (**a**–**e** Reproduced with permission from Ref. [1], © 2015, WILEY–VCH Verlag GmbH & Co. KGaA, Weinheim). **f** Schematics of a semitransparent piezoelectric strain sensor, resistive sensor, and photograph of their applied form on skin (*inset*). **g**, **h** Schematics of **g**, the wearable piezoelectric strain sensor and **h** resistive temperature sensor. **i** X-ray photoelectron spectroscopy results obtained from a ZnO nanomembrane (*top*) and ZnO/SWNT composite (*bottom*). **j** Output voltage of the piezoelectric strain sensor consisting of ZnO (*black*), Cr/ZnO (*red*), and Cr/ZnO/SWNT (*blue*) for repetitive bending and relaxing of the sensor. **k** E_C_ − E_F_ of the AZO nanomembrane with different concentrations of AgNPs. **l** Relative change in the resistance of various types of temperature sensors with respect to temperature. (**f**–**l** Reproduced with permission from Ref. [46], © 2015, WILEY–VCH Verlag GmbH & Co. KGaA, Weinheim)

In several cases, electronic materials having a relatively poor performance are incorporated owing to the limited processing temperature and chemical/mechanical resistance of plastic substrates [65]. Appropriately chosen functional nanomaterials compensate for these limitations and improve the device performance [46, 66]. Figure 4a shows a transparent piezoelectric motion sensor and electrotactile stimulator (inset) conformally laminated onto the human skin. The piezoelectric motion sensor consists of GP layers as the transparent electrodes, polylactic acid (PLA) as the piezoelectric material, and SWNTs as the piezoelectric performance enhancer (Fig. 4b, left). Moreover, the electrotactile stimulator utilizes doped GP layers as transparent electrodes and silver nanowires (AgNWs) as a conductivity enhancer (Fig. 4b, right). The strain-induced charge separation in PLA is the main mechanism for piezoelectric energy generation. The local increase in the modulus by the CNTs increases the locally induced strain inside PLA under deformation, which maximizes charge generation (Fig. 4c). Figure 4d shows the conductivity enhancement by AgNWs sandwiched between GP layers. The enhanced conductivity of the GP/AgNWs/GP hybrid contributes to effective electrotactile stimulation (Fig. 4e).

Figure 4f shows an illustration and optical image (inset) of a semitransparent piezoelectric strain sensor and resistive temperature sensor for measuring wrist motions and body-temperature changes for wheelchair control and hypothermia diagnosis, respectively. The strain sensor consists of a ZnO nanomembrane as the piezoelectric material and SWNT networks as the performance enhancer (Fig. 4g). The temperature sensor consists of silver nanoparticles (AgNPs) embedded in the ZnO:Al (AZO) nanomembrane for improving its sensitivity (Fig. 4h). For the strain sensor, co-deposited Cr and SWNTs layers improve the crystallinity of ZnO and passivate intrinsic defects, respectively (Fig. 4i). These modifications dramatically amplify the piezoelectric voltage output of the intrinsic ZnO nanomembrane (Fig. 4j). For the temperature sensor, E_C_ – E_F_ (E_C_, minimum energy of the conduction band; E_F_, Fermi energy level) is proportional to the concentration of AgNPs inside the ZnO nanomembrane (Fig. 4k). The high concentration of AgNPs increases the carrier density and therefore improves the sensitivity of the temperature sensor (Fig. 4l). A more in-depth study of functional hybrid nanomaterials would provide new opportunities for high-performance wearable devices.

### Wearable memories

Data recorded by wearable sensors should be either transferred or stored for the analysis. Usually, the data are stored in memory devices and retrieved when needed. In this section, two types of ultrathin deformable nonvolatile memory devices—charge-trap floating-gate memory (CTFM) [48] and resistive random access memory (RRAM) [41]—are described.

#### Deformable charge-trap floating-gate memory

**Fig. 5:**
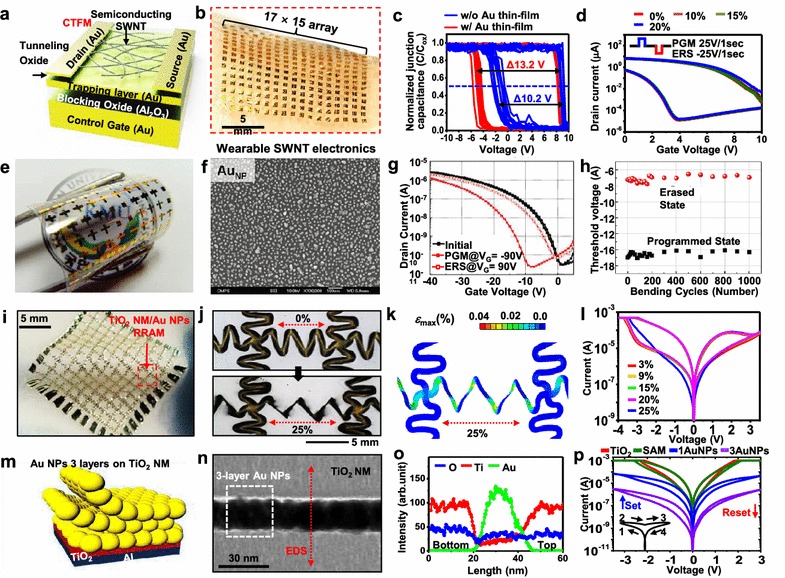
Nanomaterials embedded wearable memory devices. **a** Schematic of CTFM. **b** Photograph of the CTFM array conformally attached to human skin. **c** C–V hysteresis curves of a CNT-based memory capacitor with (*red*) and without (*blue*) an Au thin-film floating gate. **d** Transfer curves of stretched (0–20 %) CTFM for the program and erase modes. (**a–d** Reproduced with permission from Ref. [48], © 2015, American Chemical Society). **e** Photograph of a flexible organic memory device with an AuNP floating gate. **f** SEM image of AuNPs attached to the blocking oxide. **g** Transfer curves of the flexible organic memory device for the initial, programmed, and erased states. **h** Threshold voltage of the flexible organic memory device for the programmed and erased states according to the number of bending cycles. (**e**–**h** Reproduced with permission from Ref. [71], © 2010, American Chemical Society). **i** Photograph showing wearable RRAM attached to a medical bandage. **j** Optical images of wearable RRAM in the initial (*top*) and stretched (*bottom*) states. **k** Finite-element-analysis results showing the strain distribution of stretched (25 %) wearable RRAM. **l** I–V characteristic curves of wearable RRAM for different stretched states. **m** Schematic structural view of three layers of AuNPs assembled on a TiO_2_ nanomembrane, **n** TEM image showing three layers of AuNPs embedded between TiO_2_ nanomembranes. **o** Energy-dispersive X-ray spectroscopy results showing the quantitative material composition scanned along the *red arrow* in Fig. 5n. **p** I–V characteristic curves showing the bipolar switching of wearable RRAM for different trap materials. (**i**–**p** Reproduced with permission from Ref. [41], © 2014, Nature Publishing Group)

Since the concept of memory devices using floating gates was first proposed [67], field-effect transistor (FET)-based CTFM has established itself as a dominant data storage device owing to its small area and compatibility with the CMOS process [68, 69]. For the realization of deformable CTFMs as next-generation devices, the rigid active materials are replaced with deformable ones such as organic materials [70, 71], SWNTs [48], 2D nanomembranes [72], and even inorganic SiNMs [73]. Figure 5a and b show the device structure of an SWNT-based CTFM and its laminated form on the human skin, respectively. The Au nanomembrane as a floating gate maximizes the charge capturing functionality (Fig. 5c). Soft active layers of SWNT networks are located at the neutral mechanical plane and allow stable operation under deformation.

The floating gate of a continuous metal film has a critical limitation for the retention time [74]. Instead, metal nanoparticles (NPs) are a promising candidate as the floating gate to realize a fast program/erase speed and long retention time [74]. Figure 5e shows an optical image of a fabricated flexible CTFM using poly(4-vinylphenol) (PVP), pentacene, and gold nanoparticles (AuNPs) as the dielectric, semiconductor, and charge-trap layer, respectively. AuNPs are electrostatically adsorbed onto the PVP blocking oxide, thereby forming a monolayer of AuNPs (Fig. 5f). A large on/off window (>10 V) is obtained owing to the high density of AuNPs (Fig. 5g). Repetitive bending up to 1000 cycles with a bending radius of 20 mm does not diminish the performance of the CTFM.

#### Nanoparticle-embedded wearable RRAM

RRAM is another promising candidate for future nonvolatile memory devices [75–77]. By integrating RRAM with wearable sensors, a low power consumption and mechanical deformability are important for long-term use in mobile environments [41]. Figure 5i shows wearable RRAM consisting of AuNP charge-trap layers that reduce its operation current. Serpentine interconnections make the wearable RRAM stretchable up to 25 % strain (Fig. 5j–l). AuNPs embedded between TiO_2_ nanomembranes by Langmuir–Blodgett assembly form a uniform layer over a large area (Fig. 5m–o). The operation current of the wearable RRAM with one AuNP layer is decreased by one order of magnitude compared to that without AuNPs (Fig. 5p). Three layers of AuNPs exhibit a larger current decrease (by almost a factor of three).

### Wearable displays

To construct user-interactive wearable electronic systems, deformable displays that visualize measured or stored data are indispensable for users. Recently, several breakthroughs in deformable LED technologies, including deformable inorganic/organic LEDs [51, 78–81], polymer LEDs [82–84], and quantum-dot LEDs (QLEDs) [85–87], have been reported.

**Fig. 6:**
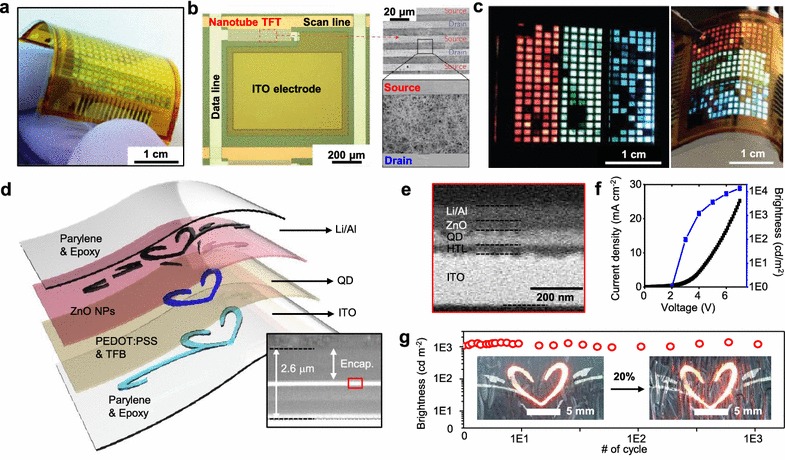
Deformable displays. **a** Photograph of a fabricated flexible OLED display containing 16 × 16 pixels. **b** Optical image of one pixel of the flexible OLED (*left*) and an enlarged view of the multiplexing CNT FET (*right*). **c,** Full-color flexible OLED display in which all pixels are turned on (*left*) and its bent form (*right*). (**a**–**c** Reproduced with permission from Ref. [50], © 2013, Nature Publishing Group). **d** Schematic exploded view showing the device structure of a wearable QLED and an SEM image showing the cross section of the display. **e** TEM image of the active layer indicated by the *red* *box* in the *inset* of **d**. **f** J–V–L characteristic curves of the wearable QLED. **g** Stable brightness of the wearable QLED during repetitive stretching cycles. Insets show the initial and stretched states of the wearable QLED. (**d**–**g** Reproduced with permission from Ref. [44], © 2015, Nature Publishing Group)

Figure 6a–c show an image of a deformable actively multiplexed organic LED array, the device structure, and the bending capability, respectively. However, organic light-emitting materials have a low stability in air and a low photostability, and thus they need thick encapsulation under ambient conditions. Quantum dots, on the other hand, have favorable properties such as a good stability in air, good photostability, printability on various substrates, and a high brightness at low operating voltages, which are important key factors for deformable/wearable displays [88, 89]. Figure 6d and e show the structure of recently reported wearable QLED devices [44]. Thanks to ultrathin active and encapsulation layers, the total thickness of the device is ~ 2.6 μm, enabling conformal contact with the wearer’s skin. The wearable QLED is turned on at a low voltage (2 V; Fig. 6j) and endures 20 % stretching up to 1000 cycles without any degradation in its brightness (Fig. 6g). The use of biocompatible quantum dots and the replacement of the rigid transparent electrodes with soft ones further improve the practical applications of wearable QLEDs.

### Wearable energy devices

Energy storage devices and power generators that supply power to wearable electronics need flexibility and biocompatibility. An all-solid-state supercapacitor (SC) [45, 49, 90, 91] is a suitable energy storage device with regard to this point. Moreover, SCs have a high power density, a fast charging/discharging speed, and cycle durability [92]. In case of the wearable power generators, flexible and soft fiber-based materials are suitable owing to the requirement of high deformability [28]. In this section, carbon-nanomaterial-based flexible SCs and organic nanofiber-based power generators are reviewed.

#### CNT-based wearable energy devices

**Fig. 7:**
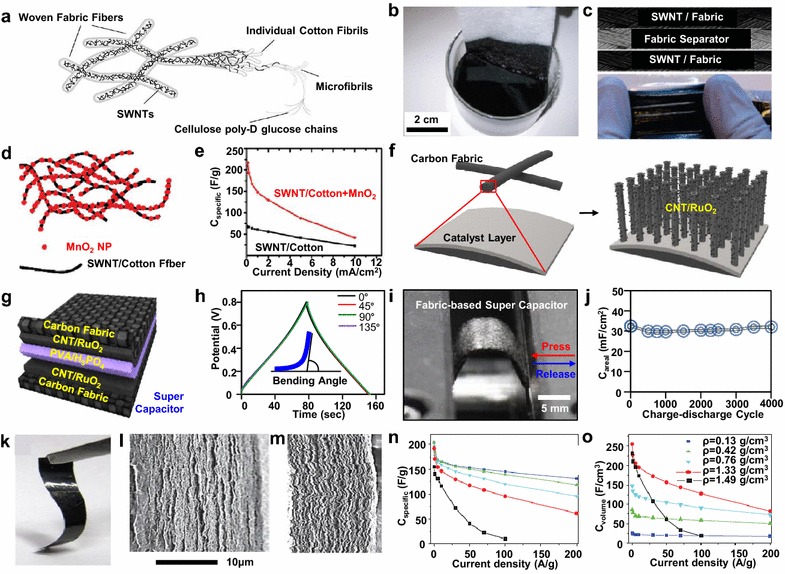
Energy storage devices based on 1D/2D carbon nanomaterials. **a** Schematic microstructure of cotton fabric with random networks of CNTs inside. **b** Photograph of the cotton fabric dipped in the CNT ink. **c** Structure of the fabric-based supercapacitor (*top*) and a photograph when it is stretched (*bottom*). **d** Schematic of the SWNT/cotton fiber electroplated with MnO_2_ nanoparticles. **e** Specific capacitances of the supercapacitors with (*red*) and without (*black*) MnO_2_ within the current density range of 0–10 mA/cm^2^. (**a**–**e** Reproduced with permission from Ref. [49], © 2010, American Chemical Society). **f** Schematic of the vertical CNT forest grown on the carbon fabric and electroplated with RuO_2_ nanoparticles. **g** Schematic exploded view of the carbon-fabric-based wearable supercapacitor. **h** Galvanostatic charge–discharge curves of the wearable supercapacitor at different bending angles. **i** Photograph showing the fabric-based supercapacitor while bent. **j** Areal capacitances of the supercapacitor for different charge–discharge cycles. (**f**–**j** Reproduced with permission from [45], © 2014, WILEY–VCH Verlag GmbH & Co. KGaA, Weinheim). **k** Photograph of the GP-stacked film. **l**–**m** SEM images of the cross sections of the GP film with **l** 78.9 and **m** 27.2 vol% of H_2_SO_4_. **n** Specific and **o** volumetric capacitances of the GP-based supercapacitors with different packing densities (ρ) for different current densities. (**k**–**o** Reproduced with permission from Ref. [15], © 2013, American Association for the Advancement of Science)

The excellent electrochemical properties, electrical conductivity, large surface area, and mechanical softness of CNTs make them apt for the electrodes and current collectors of wearable SCs [93]. Cui et al. dipped fabric into a CNT-dispersed ink to coat the fabric fibers with CNT random networks (Fig. 7a, b) [49]. These engineered fabric electrodes assembled with a fabric separator in between form the SC (Fig. 7c). The large surface area of CNTs enables further decoration with other nanomaterials (e.g., pseudocapacitive metal oxide NPs such as MnO_2_ and RuO_2_) [94, 95]. The surface of the CNT fabric is electroplated with MnO_2_ NPs (Fig. 7d, e), which increases the specific capacitance.

Instead of coating fabrics, carbon fibers are used to make a woven fabric, which can be applied to flexible textile electrodes [45]. To maximize the surface area, vertically-aligned CNTs are additionally synthesized on the carbon fabric. Electroplating vertical CNTs with RuO_2_ NPs further increases the capacitance (Fig. 7f), and an all-solid-state wearable SC is fabricated by sandwiching a poly(vinyl alcohol) (PVA)-H_3_PO_4_ gel electrolyte between two modified carbon fabric electrodes (Fig. 7g). The resulting SC exhibits high performance up to 135-degree bending and 4000 charge–discharge cycles (Fig. 7h–j).

#### GP-based wearable energy devices

Multiple chemically converted GP sheets are beneficial for fast ion transport [15]. GP flakes and/or reduced GP oxides are densely packed by capillary pressure to fabricate flexible carbon electrodes (Fig. 7k). The packing density (ρ) of the GP sheets can be controlled by changing the ratio of the volatile and nonvolatile liquids in the gel (Fig. 7l, m). Figure 7n and o show the specific capacitance and volumetric capacitance, respectively, of SCs using stacked GP electrodes for different values of ρ. The specific capacitance slightly decreases as ρ increases, whereas the volumetric capacitance is nearly proportional to ρ. Although most SCs made of activated carbon exhibit a volumetric energy density of 5–8 Wh/L, SCs made of the GP assembly exhibit a volumetric energy density of 60 Wh/L, which is similar to that of lead-acid batteries (50–90 Wh/L).

#### Organic nanofiber-based wearable power generators

To harvest electrical energy from body movements, piezoelectric nanogenerators (PENGs) and triboelectric nanogenerators (TENGs) have been used [28, 96]. Organic nanofibers such as polyvinylidene fluoride (PVDF) formed by using electrospinning processes have shown superb deformability as well as high piezoresistivity, facilitating its use in wearable applications [18, 28]. Piezoelectric power generation using a single PVDF nanofiber [97], aligned multiple PVDF nanofibers [98, 99], and randomly distributed nanofiber networks [100] have been demonstrated. Parallel and series connection of PVDF nanofibers increase the generated voltage and current [98]. However, relatively low output power of PENGs has limited the application for wearable devices with high power consumption [28]. In contrast, TENGs have shown much higher output power than PENGs [28]. Electrospun PVDF nanofibers are also suitable for fabrication of the TENG because of their strong electronegativity and high porous morphology offering large contact area to increase the output power [28, 101]. The PVDF nanofiber-based TENG has been recently demonstrated as wearable forms [28]. Seamless integration of the organic nanofiber-based wearable power generators with energy storage devices and control circuits is another important future research topic.

## Conclusions

The mechanical, electrical, and optical properties of bulk materials change as their size is reduced and/or nanoscale structure engineering is introduced. By using the unique properties of such nanomaterials or their hybrids, many breakthroughs in wearable devices have been accomplished. In this paper, we reviewed the current status of wearable devices including sensors/actuators, memory devices, displays, and energy storage devices. We particularly focused on the use of functional nanomaterials to enhancing the deformability and performance of these devices. Continuous research and development of new nanomaterials/hybrids and their integration into variety of electronic/optoelectronic devices would provide new opportunities for next-generation wearable electronics.
